# Choroidal Remodeling in Age-related Macular Degeneration and Polypoidal Choroidal Vasculopathy: A 12-month Prospective Study

**DOI:** 10.1038/s41598-017-08276-4

**Published:** 2017-08-11

**Authors:** Daniel Shu Wei Ting, Yasuo Yanagi, Rupesh Agrawal, Hwei Yee Teo, Sophia Seen, Ian Yew San Yeo, Ranjana Mathur, Choi Mun Chan, Shu Yen Lee, Edmund Yick Mun Wong, Doric Wong, Tien Yin Wong, Gemmy Chui Ming Cheung

**Affiliations:** 1 Singapore Eye Research Institute, Singapore National Eye Center 11 Third Hospital Avenue, Singapore, 168751 Singapore; 2 Duke-NUS Medical School, Singapore, 168751 Singapore; 3 National Healthcare Group Eye Institute, Department of Ophthalmology, Tan Tock Seng Hospital, Singapore, Singapore; 4 Nanyang Technology University, Singapore, Singapore; 5 Yong Loo Lin School of Medicine, National University of Singapore, Singapore, Singapore

## Abstract

Choroid thinning occurs in age-related macular degeneration (AMD). However, it remains unclear whether the reduction is due to reduction in choroidal vessels or shrinkage of choroidal stroma, or both. The purpose of this study was to evaluate the changes of the choroidal vascular and stromal area in 118 patients with typical AMD (t-AMD) and polypoidal choroidal vasculopathy (PCV) over a 12-month period. We used spectral-domain optical coherence tomography (SD-OCT) with enhanced depth imaging (EDI) mode to measure the subfoveal choroidal thickness (CT), central retinal thickness (CRT) and choroidal vascularity index (CVI - ratio of luminal area to total choroidal area). At baseline, PCV eyes had higher CRT (471.6 µm vs 439.1 µm, p = 0.02), but comparable subfoveal CT and CVI, compared to t-AMD. Eyes with high CVI at baseline showed marked reduction in stromal area compared with eyes with average or low CVI. Over 12 months, CRT and subfoveal CT significantly decreased (p < 0.001) in both subtypes. Eyes with high baseline CVI showed significant CVI reduction from baseline to month 12 (p < 0.001), whereas eyes with average to low baseline CVI showed increase in CVI. These differences in choroidal vascularity may reflect different predominant pathogenic processes and remodeling in AMD eyes with varying spectrum.

## Introduction

Age-related macular degeneration (AMD) is a major cause of vision impairment globally^1–3^. Advances in imaging technology such as enhanced depth imaging (EDI) and swept source OCT (SS-OCT) have enabled the quantitative and qualitative study of choroidal features and better our understanding of the potential role of choroid in the pathogenesis of AMD^4^.

Pachychoroid phenotype is a relatively new entity. The initial description of pachychoroid related mainly to choroidal thickening observed on OCT. Recently, additional qualitative features have been included, such as the presence of pathologically dilated outer choroidal vessels, focal attenuation of choriocapillaries and Sattler layers, regional choroidal hyperpermeability^5–7^. An eye with pachychoroid may also have normal or reduced choroidal thickness with dilated choroidal vessels and atrophy of choriocapillary layer^8^. This description, however, remains largely descriptive and is subjected to variations in individual interpretation. In order to quantify the structural change in choroid, the term choroidal vascularity index (CVI) has recently been coined^9, 10^. This method utilizes the binarization of the choroidal layer to differentiate the vascular area (luminal area) from the interstitial area (stromal area). We have previously established normative CVI in a population study^11^ and reported that CVI is reduced in patients with AMD^10^. In a recent paper, we have also reported that this binarization technique could differentiate PCV into two subtypes according to choroidal vascularity^12^.

Reduction in CT has been observed in eyes treated with anti-VEGF and PDT^9, 13^, but it remains unclear whether the reduction is due to reduction in choroidal vessels or shrinkage of choroidal stroma, or both. To address this gap, the purpose of this study is to evaluate the changes of the choroidal vascular and stromal area in patients with t-AMD and PCV over a 12-month period, after receiving treatment. We hypothesize that there is a possible choroidal remodeling in eyes with t-AMD and PCV, CVI would decrease in association with CT if there is preferential loss of choroidal vasculature, and that eyes with pachychoroid features (showing high baseline CVI) may exhibit a more marked reduction in CVI.

## Methods

### Study Population

This was a prospective, observational cohort study involving treatment naïve patients with typical AMD (t-AMD) and PCV in the Asian AMD Phenotyping Study^14^. Detailed methodology had been published elsewhere^14, 15^. This study was approved by the Centralized Institutional Review Board (CIRB) of SingHealth, Singapore (protocol number R697/47/2009) and conducted in accordance with the Declaration of Helsinki. The datasets generated during and/or analysed during the current study are available from the corresponding author on reasonable request.

After informed consent, all patients underwent comprehensive ophthalmic evaluation including best-corrected visual acuity (BCVA) testing using logarithm of the minimum angle of resolution (logMAR), refraction, slit-lamp biomicroscopy examination, and imaging according to standardized protocol which included color fundus photography, spectral domain optical coherence tomography spectral domain OCT (Heidelberg Retina Angiography, Spectralis; Heidelberg Engineering, Heidelberg, Germany) and angiography with fluorescein and indocyanine green (TRC-50X/IMAGEnet 2000, Topcon, Tokyo, Japan or Spectralis; Heidelberg Engineering, Heidelberg, Germany).

The diagnosis of t-AMD and PCV were made based on fundus fluorescein angiogram (FFA) and indocyanine green angiogram (ICGA). The t-AMD is diagnosed based on presence of choroidal neovascularization (CNV)^16^, while PCV is diagnosed when one of the following is present: (1) Protruded orange-red elevated lesions observed by fundus examination; and/or (2) characteristic polypoidal lesions seen in ICG^17–19^.

We followed up all patients prospectively according to clinical need, but minimum reviews, including BCVA and OCT imaging were repeated at month 3, 6 and 12. For this study, we only included all eyes that completed the 12-month follow-up and had OCT gradable for CVI assessment at both time-points. We also recorded the central retinal thickness (CRT). All t-AMD eyes were treated with intravitreal anti-VEGF based on a pro-re-nata (PRN) regimen. Retreatment with additional anti-VEGF injections was made in the presence of persistent or recurrent subretinal or intraretinal fluid or hemorrhage. In cases of PCV, anti-VEGF was recommended where there was significant fluid or hemorrhage subfoveally. In addition, photodynamic therapy (PDT) was added for extensive PCV lesions^20^.

### Choroidal Thickness Measurement

In this study, we included patients who had spectral domain OCT with enhanced depth imaging mode. A minimum of 25 B-scans per volume scan of 20° × 20° was used. Each scan was averaged with 9 frames per B-scan with a distance of 240 µm between each B-scan, centering at the fovea.

**Figure 1:**
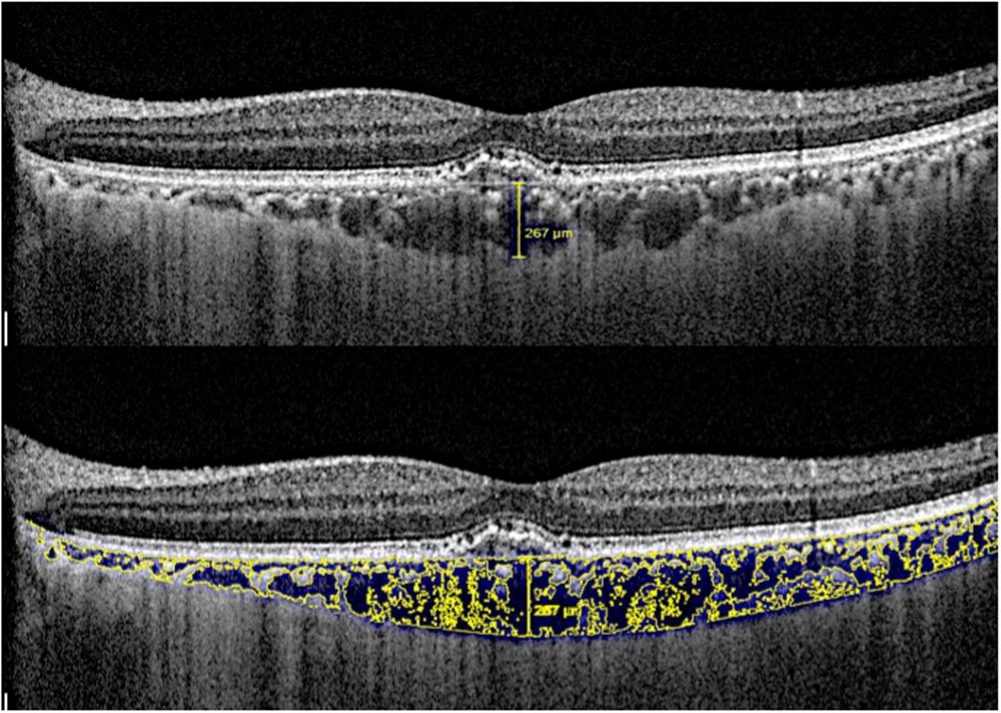
The measurement of subfoveal choroidal thickness (top) and choroidal vascularity index (bottom) using image binarization technique and Image J.

Two trained graders, masked to the clinical information, measured the choroidal thickness independently at the subfoveal region. We defined choroidal thickness as the distance between the hyper-reflective line corresponding to Bruch’s membrane beneath the retinal pigment epithelium and choroidal-scleral interface, as used in previous studies (Fig. 1)^21^.

### Image binarization

We utilized the entire length of EDI OCT scan for image binarization described in our previous study^10^. Using the public domain software Image J^22^, a trained grader selected total choroid area (TCA) and added in the ROI manager, using a polygon tool. After converting the image into 8 bit, Niblack auto local thresholding was subsequently applied which gives the mean pixel value with standard deviation for all the points. After applying the colour threshold, the Luminal Area (LA) was highlighted and inserted onto the ROI manager. To determine the LA, both the areas (TCA and LA) in ROI manager were selected and merged by AND operation. The composite third area was added to the to the ROI manager. The first area represents the total of the choroid selected, and the third composite area is the vascular or LA. The CVI was calculated by dividing LA by TCA (Fig. 1).

### Statistical Analysis

The primary outcome was the change in subfoveal choroidal thickness and CVI from baseline to month 12. We analyzed the categorical variables using paired t-test while the generalized linear mixed models were utilized to assess the effect of anti-VEGF on choroidal thickness and best corrected visual acuity at month 3, 6 and 12. We also stratified our analysis based on diagnosis of typical AMD versus PCV. For all eyes, we subdivided the groups into tertiles according to the baseline CVI – upper, mid and lower tertiles, in order to investigate the choroidal vascular changes in pachy- versus non-pachychoroid eyes. For PCV eyes, we further subdivided the eyes into monotherapy (anti-VEGF alone) versus combination therapy (anti-VEGF and PDT). All data were expressed as mean +/− standard deviation, and a p-value < 0.05 was considered to be statistically significant. All statistical analyses were performed using SPSS version 21 (SPSS, Chicago, IL, USA).

## Results

We included 118 eyes (55 had t-AMD, 63 had PCV) from 118 patients. The baseline characteristics are summarized in Table 1. At baseline, there was no difference in terms of age, gender, smoking status, spherical equivalence, BCVA, CRT, subfoveal choroidal thickness, CVI, TCA, LA and SA between AMD and PCV eyes (Table 1).

**Table 1:** Patients’ demographics, central retinal thickness, choroidal thickness (affected and fellow eye) and choroidal vascularity index (CVI) for typical AMD (t-AMD) and PCV patients.

Affected eyes	Overall (n = 118)	t-AMD (n = 55)	PCV (n = 63)	P value (t-AMD vs PCV)
Age mean, (SD)	69.7 (9.99)	69.9 (10.3)	69.2 (9.81)	0.90
Sex, female (n,%)	54, 45.8%	26, 47.3%	28, 44.4%	0.85
Smoking status (n, %)	20, 17.4%	12, 21.8%	8, 12.7%	0.33
Spherical equivalent (mean, SD)	−0.28 (2.24)	−0.37 (2.55)	−0.20 (1.91)	0.69
Best-corrected VA (logMAR) (mean, SD)	0.812 (0.546)	0.803 (0.568)	0.820 (0.531)	0.86
Central Retinal thickness	457.0 (179.2)	439.1 (123.2)	471.6 (215.3)	0.31
Subfoveal choroidal thickness	237.2 (87.1)	231.2 (87.9)	242.5 (86.8)	0.48
Choroidal vascularity index, CVI (%)*	62.0 (2.9)	62.3 (2.8)	61.9 (3.1)	0.97
Total choroidal area, TCA (mm^2^)*	3.69, (1.09)	3.63 (1.09)	3.71 (1.16)	0.84
Luminal area, LA (mm^2^)*	2.26 (0.70)	2.24 (0.67)	2.29 (0.70)	0.89
Stromal area, SA (mm^2^)*	1.41 (0.47)	1.40 (0.44)	1.42 (0.68)	0.91

^*^CVI, TCA, LA and SA were calculated based on 98 eyes (47 t-AMD and 51 PCV), due to exclusion of 20 eyes that were ungradable.

We excluded 20 eyes (8 t-AMD; 12 PCV) from the longitudinal analysis due to large haemorrhagic retinal pigment epithelial detachments, resulting in non-gradable CVI at baseline. Most patients (72.4%, n = 71) had intravitreal bevacizumab, 17 eyes (17.3%) had ranibizumab and 10 (10.2%) had aflibercept. Over a 12-month period, BCVA improved from 0.83 to 0.55 (p = 0.02) for t-AMD and 0.78 to 0.61 (p = 0.13) for PCV eyes (Table 2). CRT decreased significantly from baseline to month 12 for t-AMD (438.3 to 353.3, p < 0.001) and PCV eyes (472.1 to 317.4, p < 0.001). Similarly, subfoveal CT decreased significantly from baseline to month 3, month 6 and month 12 for both t-AMD and PCV (t-AMD: 226.7, 198.1, 189.8, 184.0; PCV: 237.6, 212.5, 208.4, 189.0, p < 0.001).

**Table 2:** The change in best-corrected visual acuity (BCVA), central retinal thickness, subfoveal choroidal thickness and choroidal vascularity index from baseline to month 3, 6 and 12 for eyes with typical AMD or PCV completing 12-month study.

Typical AMD (n = 47)	Baseline Mean, SD	Month 3 Mean, SD	P value (month 3 vs baseline)	Month 6 Mean, SD	P value (month 6 vs baseline)	Month 12 Mean, SD	P value (month 12 vs baseline)
Best-corrected visual acuity, LogMAR	0.833, 0.542	0.690, 0.501	<**0**.**01**	0.666, 0.533	**0**.**022**	0.548, 0.512	**0**.**025**
Central retinal thickness, µm	438.3, 122.9	345.3, 131.7	<**0**.**001**	370.8, 126.5	**0**.**0072**	353.3, 136.7	<**0**.**001**
Subfoveal choroidal thickness, µm	226.7, 92.1	198.1, 81.4	<**0**.**001**	189.8, 85.1	<**0**.**001**	184.0, 48.1	<**0**.**001**
Choroidal vascular index (CVI), %	62.3, 2.8	63.5, 3.3	0.316	63.4, 3.5	0.222	63.5, 3.5	0.391
Total choroidal area (TCA)	3.63, 1.09	2.81, 0.88	<**0**.**001**	2.84,0.86	<**0**.**001**	2.86, 0.88	<**0**.**001**
Luminal area (LA)	2.24, 0.67	1.76, 0.58	<**0**.**001**	1.80, 0.57	<**0**.**001**	1.78, 0.56	<**0**.**001**
Stromal Area (SA)	1.40, 0.44	1.05, 0.32	<**0**.**001**	1.04, 0.31	<**0**.**001**	1.09, 0.35	<**0**.**001**
Anti-VEGF injections (mean cumulative)		2.24, 1.10		3.11, 1.00		4.28, 1.48	
**PCV (n = 51)**							
Best-corrected visual acuity, LogMAR	0.78, 0.54	0.674, 0.586	0.422	0.683, 0.581	0.283	0.614, 0.542	0.125
Central retinal thickness, µm	472.1, 219.8	307.8, 121.4	<**0**.**001**	304.8, 110.6	<**0**.**001**	317.4, 115.7	<**0**.**001**
Subfoveal choroidal thickness, µm	237.6, 85.6	212.5, 84.0	<**0**.**01**	208.4, 91.8	<**0**.**001**	189.0, 48.5	<**0**.**001**
Choroidal vascular index (CVI), %	61.9, 3.1	63.2, 3.1	0.100	63.0, 3.5	0.213	62.5, 3.7	0.350
Total choroidal area (TCA)	3.71, 1.16	3.14, 1.07	<**0**.**001**	2.95, 1.00	<**0**.**001**	2.99, 0.99	<**0**.**001**
Luminal area (LA)	2.29, 0.70	1.99, 0.72	<**0**.**001**	1.87, 0.66	<**0**.**001**	1.87, 0.68	<**0**.**001**
Stromal Area (SA)	1.42, 0.48	1.15, 0.37	<**0**.**001**	1.08, 0.36	<**0**.**001**	1.12, 0.33	<**0**.**001**
Anti-VEGF injections (mean cumulative)		2.36, 0.87		3.34, 1.13		3.92, 1.08	
PDT injections (mean cumulative)		0.37, 0.53		0.46, 0.62		0.49, 0.67	

All eyes had complete baseline, month 3, month 6 and month 12 data.

CVI in t-AMD eyes increased from baseline to month 3 (62.3% to 63.5%, p = 0.32) and remained largely unchanged till month 12, while CVI in PCV eyes increased from baseline to month 3 (61.9% to 63.2%, p = 0.10), remained the same at month 6 and decreased at month 12 (62.5%), though the change were not statistically significant. TCA, LA and SA for both t-AMD and PCV decreased significantly over the first 3 months and showed little further change till month 12 (Table 2). The mean number of anti-VEGF injections was 4.28 (±1.48) and 3.92 (±1.08) for AMD and PCV, respectively during the first year of treatment. Twenty-four eyes with PCV also received PDT treatment in addition to anti-VEGF therapy.

**Figure 2:**
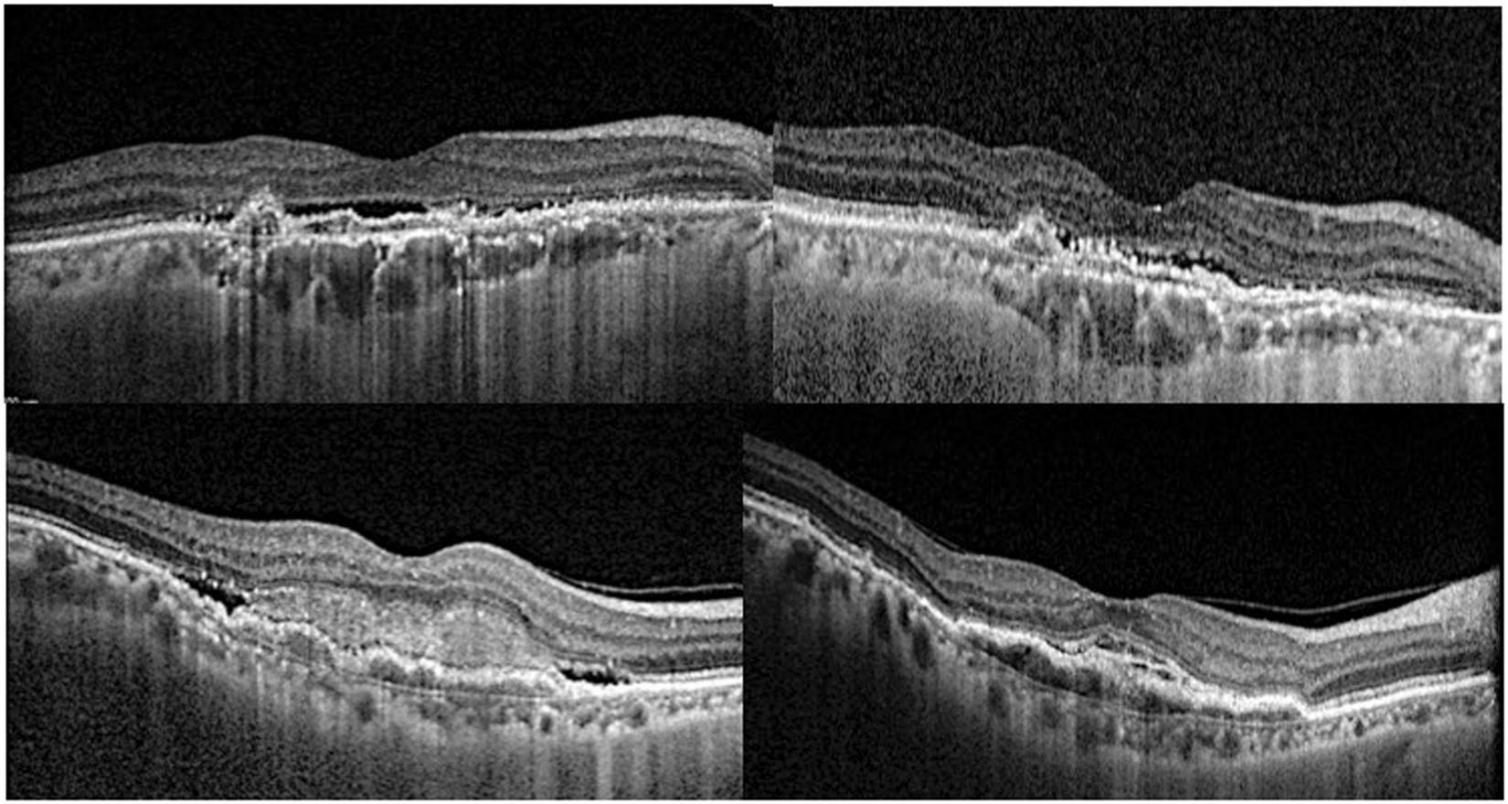
The choroidal vascularity index (CVI), total choroidal area (TCA), luminal area (LA) and stromal area (SA) changes of neovascular age-related macular degeneration eyes with upper and lower CVI tertile from baseline to month 12. (**A**, top left) Baseline OCT in an eye within the highest baseline CVI tertile (CVI: 67.8%; LA: 2.10 mm^2^; SA: 1.0 mm^2^). Dilated Haller’s layer vessels and relative compression of choroidal stroma can be appreciated. At month 12 (**B**, top right), there was reduction in CVI and luminal area (CVI: 58.4%; LA: 1.45 mm^2^; SA: 1.18 mm^2^. (**C**, bottom left) Baseline OCT in an eye within the lowest baseline CVI tertile (CVI: 57.7%; TCA: 2.98 mm^2^; LA: 1.72 mm^2^; SA: 1.26 mm^2^). The Haller’s layer vessels are less dilated compared to (**A**) that lies within the highest tertile. At month 12 (**D**, bottom right), there was an increase in CVI due to significant reduction in SA compared to LA (CVI: 65.3%; TCA: 2.41 mm^2^; LA: 1.57 mm^2^; SA: 0.84 mm^2^).

We further examined the changes within the choroid by stratifying eyes according to baseline CVI into tertiles. At baseline, eyes in the highest CVI tertile exhibit markedly lower SA (1.24 mm^2^ vs 1.58 mm^2^, p < 0.001) and only marginally increased LA (2.30 mm^2^ vs 2.25 mm^2^, p = 0.74) compared to eyes in the lowest tertiles. Over the 12-month follow-up period, we observed that CVI was significantly reduced in eyes with the highest CVI tertile (65.3% vs 62.4%, p < 0.001) (Table 3). Closer examination of choroidal sub-components revealed that the reduction in CVI was the result of a more pronounced reduction in LA (−18.5%) compared to SA (−9.2%). (Figure 2A and B) In contrast, eyes with baseline CVI in the mid and lowest tertiles showed an increase in CVI from baseline to month 12, resulting from more pronounced reduction in SA (−23.0% and −33.5%) compared to LA (−26.9% and −16.9%) (Fig. 2C and D).

**Table 3:** The change of choroidal vascularity index, luminal area, stromal area and subfoveal choroidal thickness in tertiles between baseline and month 12 (M12) for neovascular AMD (t-AMD and PCV) eyes.

CVI Tertiles	Choroidal vascularity index (CVI)				Luminal area				Stromal area				Subfoveal choroidal thickness			
	Baseline	M12	% change	p value	Baseline	M12	% change	p value	Baseline	M12	% change	p value	Baseline	M12	% change	p value
T1 (lowest)	59.01 (1.22)	60.79 (3.43)	+3.0	0.01	2.25 (0.71)	1.64 (0.51)	−26.9	<0.001	1.58 (0.50)	1.04 (0.26)	−33.5	<0.001	236.3 (87.8)	178.2 (55.8)	−24.59	<0.001
T2 (mid)	61.78 (0.71)	64.03 (2.81)	+3.6	0.12	2.29 (0.65)	1.91 (0.58)	−16.9	<0.001	1.42 (0.39)	1.01 (0.40)	−29.3	<0.001	234.4 (82.89)	190.0 (57.3)	−18.98	<0.001
T3 (highest)	65.25 (1.86)	62.37 (3.87)	−4.4	<0.001	2.30 (0.65)	1.88 (0.60)	−18.5	0.01	1.24 (0.39)	1.13 (0.37)	−9.2	0.23	236.1 (82.7)	189.3 (57.1)	−19.83	0.01
P trend	<0.001	0.08			0.72	0.09			<0.001	0.32			0.99	0.43

We also evaluated whether combination of anti-VEGF with PDT affected the changes in choroid in different ways than anti-VEGF monotherapy. There was a significant improvement in BCVA and reduction in subfoveal CT from baseline to month 12 in both treatment groups. CVI, however, showed no significant change in either group, although there was small numerical reduction in the combination group (63.1 to 61.2%, p = 0.54) but not in the anti-VEGF monotherapy group (61.2 to 62.6%, p = 0.83) (Table 4). In a multivariable logistic regression which included age, gender, smoking, diagnosis (t-AMD vs PCV), baseline CT, baseline CVI, PDT and total injection number, only baseline CVI was found to significantly influence change in CVI over 12 months (p < 0.001).

**Table 4:** The changes in best-corrected visual acuity (BCVA), choroidal vascularity index (CVI) and subfoveal choroidal thickness of PCV eyes according to treatment groups.

	Baseline	Month 3	Month 6	Month 12	P
	Mean, SD	Mean, SD	Mean, SD	Mean, SD	trend
**PCV with monotherapy** (**n** = **27**)					
i. Best-corrected VA	0.894, 0.579	0.733, 0.697	0.697, 0.620	0.620, 0.522	**0.01**
ii. Choroidal vascular index (%)	61.2, 2.8	63.6, 3.2	63.3, 2.7	62.6, 4.3	0.83
iii. Subfoveal choroidal thickness (µm)	236.6, 90.6	208.4, 83.1	199.6, 87.2	222.8, 97.0	**0.04**
**PCV with combination therapy** (**n** = **24**)					
i. Best-corrected VA	0.777, 0.566	0.640, 0.578	0.611, 0.555	0.593, 0.541	**0.01**
ii. Choroidal vascular index (%)	63.1, 3.0	62.1, 2.8	62.2, 2.6	61.2, 2.7	0.54
iii. Subfoveal choroidal thickness (µm)	228.3, 81.2	201.2, 84.4	200.5, 93.8	203.6, 90.1	**<0.001**

## Discussion

In this prospective study, we evaluated the remodeling of the choroid by demonstrating the detailed changes within sub-components of the choroid in eyes with t-AMD and PCV. As previously described, there was a significant reduction in mean subfoveal CT from baseline to month 12 in both t-AMD and PCV groups. Despite this progressive thinning of the choroid, there was substantial improvement in vision from baseline to month 12. Interestingly, we noticed the thinning of the choroid was contributed by thinning of sub-components of the choroid to different extent, depending on baseline features. Although the CVI remained unchanged in the overall group, we observed that CVI decreased in eyes with high baseline CVI, but increased in eyes with average to low baseline CVI.

Although increased choroidal thickness has been reported in eyes with PCV, our previous work suggested a wide range of CT may be seen in eyes with PCV^13^. However, using the binarization technique similar to this study, PCV may be divided into two subtypes according to choroidal vascularity^12^. Therefore, in the present study, we focused on further evaluation of choroidal sub-components at baseline and their longitudinal changes after treatment^12^. Eyes with high baseline CVI (highest tertile) displayed markedly lower stromal area but only slightly higher luminal area compared to eyes with low baseline CVI (lowest tertile). This pattern suggests the dilated choroidal vasculature in eyes with high CVI may be accompanied by compressed or atrophied choroidal stroma, and is in keeping with previous description of pachychoroid spectrum^5, 7^. With treatment, reduction in both the luminal and stromal areas was noted, but was much more prominent in the luminal than the stromal component, resulting in a decrease in CVI noted at month 12. This observation suggests that choroidal thinning observed in pachychoroid eyes could be primarily due to reduction in vascularity within Sattler and Haller layers. We speculate that this effect may be beneficial in eyes with pachychoroid feature as the reduction in vascularity may alleviate the compression on the inner choroid, thus reducing the hypoxia or mechanical damage to the surrounding choriocapillaries. The stromal component in these pachychoroid eyes is already reduced at baseline, and showed less marked reduction to month 12.

In contrast, eyes with low baseline CVI (lowest tertile) displayed higher stromal area compared to eyes with high baseline CVI. The mechanism for such increase in stromal area is unclear, but may be due to increased permeability of the choroidal vessels resulting in exudation into the stroma, and inflammatory activities. During follow-up, there was a marked reduction in the stromal area (−33.5%) in these eyes, suggesting that treatment might have led to modulation of such exudative or inflammatory activities. Serine proteases and tissue inhibitor of metalloproteinase-3 (TIMP3) have also been shown to play a major role in regulating the balance between the fibrillar collagen and ground substance in the choroidal stroma. It will be interesting for future research to investigate whether such proteases and protease inhibitors change with AMD treatment or as lesions evolve.

There have been few studies to-date to examine the longitudinal changes in the detailed sub-components of the choroid. In a recent study by Daizumoto and coauthors^23^, they subdivided the choroidal layers into inner and outer layers at the subfoveal 1500 µm region. In 40 treatment-naïve PCV patients, they found that the inner choroid SA and outer choroid LA decreased significantly at month 3 and month 12 after intravitreal aflibercept. In our study, we analyzed the entire OCT slab (9 mm), instead of 1.5 mm subfoveal region. Both SA and LA decreased significantly at month 3, 6 and 12 (compared to the baseline). We did not subdivide the choroidal layer into inner (Sattler) versus outer (Haller) as because accurate identification of the junction between the inner/outer choroidal layers remains challenging, particularly in eyes with thin choroid. It is, therefore, difficult to conduct direct comparison between the results that arise from the two studies.

Given that this is a clinic-based prospective study, one of the limitations of our study was the lack of standardization of anti-VEGF types and use of PDT between different retinal physicians. However, we specifically examined and found no significant differences in effect on choroid in eyes treated with and without PDT (Table 4). We measured luminal and stromal areas within the 9 mm scan across the fovea. However, such global calculation may not reflect localized areas of choroidal vessel dilatation. In eyes with extensive shadowing effect from the large pigment epithelial detachment, the binarization software may not give accurate results. We therefore excluded 20 eyes with such features. Some patients did not have month 1, month 2 and month 9 OCT repeated and therefore, these additional time points were not included in our study. We did not include healthy subjects in this cohort. However, we reported normative data on CVI in a population study (65.1% ± 2.33%)^11^, and reduced CVI in eyes with exudative AMD compared to their unaffected fellow eyes (60.1% vs 62.8%, p < 0.01)^10^ in previous studies. Finally, the current measurement of CVI was based on a single horizontal cross-sectional scan across the fovea. Future software which can analyze the whole volume scan will be able to give more comprehensive information regarding the entire macula.

In conclusion, our study showed that longitudinal changes in choroidal vascular and stromal component were different and dependent on baseline choroidal vascularity. CVI is a useful quantitative tool to evaluate these detailed choroidal features, and may be more specific than choroidal thickness measurement or differentiation according to t-AMD or PCV. These differences in choroidal vascularity may reflect different predominant pathogenic processes and choroidal remodeling in eyes with and without pachychoroid and choroidal hyperpermeability.
